# Cancer treatment with kinase inhibitors: what have we learnt from imatinib?

**DOI:** 10.1038/sj.bjc.6601507

**Published:** 2004-01-06

**Authors:** D M Ross, T P Hughes

**Affiliations:** 1 Division of Haematology, Institute of Medical and Veterinary Science, PO Box 14 Rundle Mall, Adelaide SA 5000, Australia

## Abstract

Over the past few years, a number of anticancer drugs have been developed that specifically target kinases known to be oncogenic. The leading drug in this area is imatinib mesylate, which targets *ABL*, *KIT* and *PDGFR*. It has been remarkably effective in the treatment of chronic myeloid leukaemia, although resistance remains a significant problem. From the imatinib experience in this setting, we present some principles of kinase inhibition that may have more general applicability in targeted anticancer therapy. It is clear that the identification of appropriate targets (activated kinases) and monitoring levels of response (to recognise emerging resistance) are essential to optimise clinical management.

Kinases play a fundamental role in many cancers. Kinase inhibition represents a promising new anticancer strategy. The first kinase inhibitor to be developed for clinical use is imatinib mesylate (Glivec, Novartis), which was designed for use in chronic myeloid leukaemia (CML). In CML, the Philadelphia chromosome (Ph) and variants result in a translocation that codes for the chimaeric fusion protein, BCR-ABL, which is a constitutively activated kinase. Imatinib inhibits the normal Abelson tyrosine kinase (ABL) as well as BCR-ABL.

Imatinib inhibits other tyrosine kinases broadening the potential therapeutic utility to a range of neoplastic disorders (Buchdunger *et al*, 2002). The proto-oncogene *C-KIT* encodes the KIT tyrosine kinase, which serves as a receptor for stem cell factor. KIT is important in cell cycle regulation and critically important in haematopoiesis. Two receptors for platelet-derived growth factor (PDGF) are sensitive to imatinib. PDGF is involved in cell cycle regulation, angiogenesis, and fibroblast proliferation (Claesson-Welsh, 1994). Despite similarities in structure and function, PDGFR *α* and *β* are encoded on different chromosomes and differ in their affinity for individual isotypes of PDGF.

We review the current evidence regarding efficacy and safety of imatinib in Philadelphia-positive (Ph+) haematological disorders and in other disorders with evidence for imatinib sensitivity. We also review the available data on imatinib resistance. We then consider the broader implications for anticancer treatment based on kinase inhibitors.

## EFFICACY

### Chronic myeloid leukaemia

CML is a disorder of the haematopoietic stem cell consistently associated with the *BCR-ABL* fusion gene. It is characterised by the proliferation of the myeloid series, although lymphoid cells may also arise from the malignant clone. Three phases of the disease are recognised. In the chronic phase, there is indolent leukocytosis with infiltration of the liver and spleen. In the accelerated phase, there may also be acquisition of additional cytogenetic abnormalities and an increase in the proportion of immature cells in the blood or marrow. In blast crisis, which may be of lymphoid or myeloid lineage, the clinical picture resembles *de novo* acute leukaemia and carries a very poor prognosis.

### Imatinib in newly diagnosed chronic phase

Prior to the introduction of imatinib, the accepted standard therapy for newly diagnosed CML in the chronic phase was interferon *α* and cytarabine. A recently published study (O'Brien *et al*, 2003) randomised 1106 patients to interferon–cytarabine combination therapy or imatinib at a dose of 400 mg daily. With a median follow-up of 19 months, no difference in the overall survival could be demonstrated. However, crossover to imatinib therapy occurred in 318 out of 553 patients (58%) in the interferon–cytarabine group, largely due to intolerance of interferon-related side effects. The rates of complete haematologic response (CHR) (95 *vs* 56%), complete cytogenetic response (CCR: 76 *vs* 15%) and freedom from progression to accelerated phase or blast crisis (97 *vs* 92%), all showed a statistically significant improvement in the imatinib group.

### Imatinib in chronic phase after interferon failure

Patients with prior exposure to interferon are mostly in the late chronic phase (⩾12 months from diagnosis). Of 454 late chronic phase patients started on imatinib at 400 mg daily, 95% achieved CHR and 41% CCR with freedom from progression in 89% at 18 months (Kantarjian *et al*, 2002). These results for imatinib therapy are inferior to those observed in patients treated at diagnosis.

Two studies have looked at dose escalation in order to improve the response rates in this group. With imatinib treatment at 800 mg daily in interferon-failed chronic phase (Cortes *et al*, 2003), CHR was achieved in 100% and major cytogenetic response (MCR) in 90%. After 3 months, dose reduction due to toxicity had occurred in 42%, with the most common grade III–IV toxicities being haematological. Among patients started on imatinib 400 mg daily, dose escalation was an effective salvage therapy for refractoriness or relapse (Kantarjian *et al*, 2003), but the overall results with this strategy were inferior to those reported with treatment initiated at a higher dose.

### Imatinib in accelerated phase

The accelerated phase of CML typically follows several years of chronic phase and is associated with decreased response to therapy and a shortened median survival of less than 1 year. A multicentre study reporting on 181 patients treated with imatinib for accelerated phase CML suggests lower response rates and increased toxicity in comparison with the chronic phase (Talpaz *et al*, 2002). Patients were initially commenced on imatinib at 400 mg daily, but after efficacy and safety data became available, subsequent patients were treated with 600 mg daily. CHR was achieved in 53% and sustained for at least 1 month in 34%. CCR was achieved in 17%. With a median follow-up in excess of 10 months, there was a significant improvement in overall survival and freedom from progression for the higher dose group. Grade III–IV haematological toxicity occurred in 58% and severe nonhaematological toxicity was uncommon (<5%).

### Imatinib in blast crisis and Ph+ acute lymphoblastic leukaemia (ALL)

In blast crisis of CML and in *de novo* Ph+ ALL, BCR-ABL retains sensitivity to imatinib, but additional genetic abnormalities conferring drug resistance are common. In patients with relapsed or refractory Ph+ ALL or lymphoid blast crisis (Ottmann *et al*, 2002) and in myeloid blast crisis (Sawyers *et al*, 2002), CHR was achieved in around 20%. Despite good haematological response, in most cases relapse occurred early with a median survival of only 20–30 weeks. Relapse is commonly associated with emergence of mutations in the BCR-ABL kinase (Gorre *et al*, 2001; von Bubnoff *et al*, 2002).

### Other myeloid disorders

A clinical phenotype closely mimicking CML has been described in a patient carrying a *BCR-PDGFRA* fusion gene (Baxter *et al*, 2002). Response to imatinib was not tested. This, and potentially other fusion kinases, may account for the rare cases of otherwise typical CML, which lack *BCR-ABL*.

The cytogenetic abnormality t(5;12) has been described in a rare myeloproliferative disorder with leukocytosis and eosinophilia. Splenomegaly and skin infiltration may be present. The translocation is usually associated with a fusion of *TEL* (a regulatory gene that may be translocated in acute leukaemia) and *PDGFRB* (Apperley *et al*, 2002). Four patients with myeloproliferative disorder and confirmed *PDGFRB* fusion were treated with imatinib 400 mg daily. All achieved CHR after 4 weeks and cytogenetic remission after 9 months.

Idiopathic hypereosinophilic syndrome is characterised by persistent peripheral blood eosinophilia with evidence of end-organ damage (usually cardiac or sinopulmonary infiltration or neuropathy) without an identifiable cause. In a series of 16 patients with idiopathic hypereosinophilic syndrome, a novel fusion of *PDGFRA* and *FIP1L1* has been reported (Cools *et al*, 2003). Nine patients were found to have this fusion gene, with eight of these having a cryptic deletion of chromosome 4. Five of these nine patients and a further five patients without *PDGFRA-FIP1L1* fusion received imatinib treatment. The doses of imatinib ranged from 100 to 400 mg daily. CHR of at least 3 months duration was achieved in all but two patients, neither of whom carried the fusion gene. Efficacy at lower doses than in CML is supported by *in vitro* data, indicating that the IC_50_ (drug concentration required to inhibit proliferation by 50%) for imatinib of PDGFR*α*-FIP1L1 is approximately 2-log lower than for BCR-ABL.

Imatinib-responsive idiopathic hypereosinophilic syndrome without an identified molecular abnormality has also been reported elsewhere (Ault *et al*, 2002; Gleich *et al*, 2002). Gleich *et al* report on five patients treated with imatinib. Four patients achieved CHR. The starting dose was 100 mg daily and maintenance doses were as low as 200 mg per week. These results imply that another cryptic rearrangement involving PDGFR (or another imatinib-sensitive kinase) may be present in some patients.

Systemic mastocytosis is a rare myeloproliferative disorder with an increase in mast cells in the marrow and evidence of visceral and cutaneous infiltration. A syndrome related to histamine release may also occur. Imatinib inhibition of KIT phosphorylation in mast cell lines correlates with the inhibition of cellular proliferation (Zermati *et al*, 2003), suggesting that the cells are critically dependent on KIT activity. In systemic mastocytosis, the most common activating mutation in *C-KIT* D816V, occurs in the catalytic region of the enzyme, and also prevents binding of imatinib. Most cases of mastocytosis are therefore resistant to imatinib. However, patients with mutations in the juxtamembrane region of the KIT molecule may retain *in vitro* sensitivity (Zermati *et al*, 2003) and durable clinical responses to imatinib in doses of 400 mg daily or less have been demonstrated (Pardanani *et al*, 2002).

The importance of PDGF in the development of fibrosis and of KIT in haematopoiesis makes idiopathic myelofibrosis an appealing target for imatinib therapy. However, the results of treatment have been mixed. The results of a phase 2 trial of imatinib in 23 patients with idiopathic myelofibrosis (Tefferi *et al*, 2002) were disappointing. Partial responses were observed in some patients, but no clinically significant benefit was obtained and haematological toxicity was greater than that observed in the chronic phase of CML, despite use of the standard starting dose of 400 mg daily. On the other hand, Cortes *et al*. (2002) observed improvement in cell counts or reduction in the spleen size in over 50% of 18 patients treated with the same dose of imatinib.

A study using *C-KIT* antisense oligonucleotides *in vitro* suggests that normal erythropoiesis is significantly dependent on KIT function (Ratajczak *et al*, 1992). In polycythaemia rubra vera, although the expression of KIT is normal, an increased sensitivity of the receptor to its ligand, stem cell factor, has been observed (Dai *et al*, 1994). These observations provide a rationale for the trials of imatinib in this myeloproliferative disorder. Reports in the small numbers of patients report reduced phlebotomy requirement with imatinib treatment (Cortes *et al*, 2002; Silver, 2002; Jones and Dickinson, 2003).

In acute myeloid leukaemia (AML), KIT is expressed in at least two-thirds of cases, although the number with *C-KIT* mutations is much smaller. In a series of *de novo* childhood AML (Zwaan *et al*, 2002), *C-KIT* was found to be mutated in 6.4% with the imatinib-resistant D816V mutation accounting for approximately half of these. An *in vitro* study using leukaemic cells from 12 patients found an overall poor response to imatinib at pharmacological doses (Scappini *et al*, 2001), although there was significant heterogeneity between leukaemia samples. This most likely reflects that in most cases of AML, KIT is not critically involved in sustaining abnormal proliferation, unlike BCR-ABL, which is pathogenetic in CML. Similarly, among 17 KIT-positive patients with relapsed or refractory AML treated with imatinib monotherapy, there were no haematological remissions (Cortes *et al*, 2002). Despite these negative findings, imatinib-responsive AML has been reported, with some cases achieving CHR (Fischer *et al*, 2002; Kindler *et al*, 2003). The clarification of the molecular pathways involved in such cases may help identify a subset of AML that will benefit from imatinib treatment.

### Gastrointestinal stromal tumours

Constitutive activation of KIT is almost universal in gastrointestinal stromal tumours (GIST), and often associated with gain-of-function mutations in *C-KIT*. A study of material from GISTs of 48 patients revealed mutations in *C-KIT* in 92%, with the majority located in exon 11, which encodes the juxtamembrane region involved in activating dimerisation (Rubin *et al*, 2001).

Interestingly, a recent report found that among the small percentage of *C-KIT* wild-type GISTs, approximately one-third had mutations in the *PDGFRA* gene with evidence of abnormal activation of its tyrosine kinase (Heinrich *et al*, 2003). This overlap in disease phenotypes associated with mutations in different tyrosine kinases is mirrored in the myeloproliferative disorders with eosinophilia, and highlights the case for accurate genetic diagnosis.

Impressive activity of imatinib monotherapy has been demonstrated in a single study of 147 patients with unresectable or metastatic GIST (Demetri *et al*, 2002), despite refractoriness to conventional chemoradiotherapy. Partial response (reduction in tumour volume ⩾50%) was achieved in 54%, with only 14% showing evidence of disease progression at a median follow-up of 288 days. The median time to measurable response was 13 weeks. The median time to disease progression was not reached within 288 days. Within the study there was a randomisation of imatinib dose (400 *vs* 600 mg daily). There was no significant difference in the response or toxicity between the two groups. However, nine patients with disease progression on the lower dose were crossed over to the higher dose and three achieved improvement in disease control.

### Other solid tumours

The expression of KIT or PDGFR*β* has been observed in a number of solid tumours, including small cell lung cancer, thyroid cancer, ovarian and breast cancers, prostate cancer, seminoma, glioblastoma, and dermatofibrosarcoma protruberans. For some of these tumours, there is *in vitro* evidence of inhibition by imatinib (Buchdunger *et al*, 2002; Heinrich *et al*, 2002a). In contrast to CML, the inhibition of a single signalling pathway will not usually be sufficient to overcome the proliferative advantage of the tumour. Clinical efficacy data are awaited.

### Diagnosis and monitoring

Chronic myeloid leukaemia is one of very few diseases for which there is a universally identifiable, single genetic abnormality that provides an ideal target for therapy. In CML, the demonstration of *BCR-ABL* confirms suitability for therapy, as well as providing a target for disease monitoring. Diagnostic and monitoring strategies for CML and other imatinib-sensitive disorders are presented below and summarised in Table 1

**Table 1:** Diagnosis and monitoring of imatinib-sensitive disorders

**Disorder**	**Genetics**	**Diagnosis**	**Monitoring**
CML	*BCR-ABL* fusion	Karyotype, PCR	Karyotype quantitative PCR BCR-ABL mutation screening
Hyper-eosinophilic syndromes	Various involving *PDGFRA* and *PDGFRB*	Karyotype, PCR	Karyotype quantitative PCR mutation screening
Systemic mastocytosis	*C-KIT* activating mutation	KIT immunohistochemistry, *C-KIT* mutation detection	
Gastrointestinal stromal tumours	*C-KIT* activating mutation	KIT immunohistochemistry, *C-KIT* mutation detection	PET

.

Quantitative monitoring of *BCR-ABL* mRNA by reverse transcriptase PCR is used to follow response. Progressive reduction in *BCR-ABL* levels may be observed over a long period, sometimes in excess of 1 year before reaching a plateau. Increases in *BCR-ABL* levels are observed with the development of resistance to therapy (Hughes and Branford 2003). A single institution study of 120 late chronic phase (interferon resistant or intolerant) patients (Merx *et al*, 2002) monitored response to imatinib (400 mg daily) with peripheral blood quantitative real-time polymerase chain reaction (qPCR) for *BCR-ABL* mRNA. There was a strong correlation between bone marrow karyotype (% Ph+ metaphases) and the *BCR-ABL/ABL* ratio by qPCR. In addition, the level of *BCR-ABL* at 2 months was predictive of cytogenetic response at 6 months. At least in the short term, attainment of a major cytogenetic response (⩽35% Ph+ metaphases) is associated with a survival advantage. It seems likely that the kinetics of qPCR response will become a useful prognostic marker to identify high-risk individuals who may not respond well to imatinib monotherapy.

In other imatinib-responsive disorders, despite greater genetic heterogeneity, molecular diagnosis and monitoring should be the goal. At least in the case of myeloproliferative disorders (e.g. *PDGFRA-FIP1L1* fusion, Cools *et al*), there is evidence that this will be possible. Where the molecular abnormality has not been identified, there may still be a role for other biological markers of imatinib responsiveness. For example, the inhibition of abnormal phosphorylation of target molecules should potentially be measurable in any imatinib-sensitive disorder, although in the case of solid tumours availability of tumour tissue may limit monitoring options.

The functional monitoring of a subset of 64 GIST patients of Demetri *et al*. (2002) was undertaken with ^18^fluoro-deoxy-glucose positron emission tomography (PET) in addition to standard radiological imaging. All patients who achieved a response had a reduction in PET activity from baseline. PET responses occurred early (even within 24 h). Loss of response was associated with increased PET activity in all cases. Furthermore, a later report (van den Abbeele *et al*, 2002) showed that PET response within the first month of imatinib therapy was a strong predictor of progression-free survival over the subsequent 16 months of follow-up.

Preliminary data on the genetic prediction of imatinib response in GIST were presented by Heinrich *et al*. (2002b). They undertook mutation detection in *C-KIT* in 121 cases of unresectable GIST and correlated these findings with the clinical course of the disease. The presence of mutations in exon 11 predicted both significantly higher rates of response to imatinib (72 *vs* 32%) and longer time to treatment failure. In contrast, in an earlier study, in patients treated without imatinib, exon 11 mutation was found to be an independent adverse prognostic factor in multivariate analysis (Singer *et al*, 2002). In the future, the detection of specific *C-KIT* mutations may be used to guide imatinib dosing or combination therapy in GIST patients.

### TOXICITY

Typically, in CML at the commencement of imatinib therapy, haematopoiesis is almost exclusively derived from the *BCR-ABL* positive clone. Inhibition of this clone will inevitably result in cytopenia until there is a recovery of the suppressed residual normal haematopoiesis. Clinical trials in the different phases of CML indicate an increase in haematological toxicity with more advanced disease. Doses beyond 600 mg daily appeared to be associated with increased toxicity. Even with higher rates of cytopenia, dose-limiting and fatal toxicities were relatively uncommon.

In contrast, in GIST there was little evidence of an effect on normal haematopoiesis, as grade III–IV thrombocytopenia did not occur and grade IV neutropenia was reported in only 5%. The rates of nonhaematological toxicity appear similar in all trials for both CML and GIST and the drug is generally well-tolerated.

While the short-term side effects of imatinib are generally well-documented, the potential longer-term consequences of kinase blockade remain uncertain. Extrapolation from observations in animal models is difficult as inhibition of kinase function in a mature animal will clearly not produce the range of developmental anomalies seen in germline knockout studies. In addition, some tyrosine kinase-dependent defects may become significant only in stressed conditions, such as inflammation and tissue repair (Van Etten, 1999). However, in knockout mice there is evidence of osteoporosis and defective osteoblast maturation (Li *et al*, 2000); defects in spermatogenesis (Guerif *et al*, 2002); abnormal lymphoid proliferation and differentiation, and abnormal melanin metabolism (Van Etten, 1999; Hou *et al*, 2000). This latter observation is significant in view of clinical reports of hypopigmentation in patients receiving imatinib (Saikia *et al*, 2002; Hasan *et al*, 2003). Continuing surveillance for delayed effects of kinase blockade will be important.

## IMATINIB RESISTANCE

Resistance has been studied extensively in CML, where acquired imatinib resistance is defined as a loss of established haematologic or cytogenetic response to imatinib, as opposed to primary resistance, in which no response is achieved. In CML, acquired resistance is usually associated with restoration of BCR-ABL kinase activity (Gorre *et al*, 2001). Data on resistance in other imatinib-responsive disorders are limited at present, but it is likely that the patterns emerging in CML will be applicable to other disease models.

### What is the mechanism of drug resistance?

Three broad mechanisms may result in the restoration of kinase activity: (1) decreased intracellular levels of imatinib; (2) increased expression of the kinase; or (3) intrinsic changes in the kinase that affect its drug interaction or kinase activity.

MDR1 overexpression causing drug efflux (Mahon *et al*, 2003) has been shown to cause imatinib resistance in Ph+ cell lines *in vitro*. The binding of imatinib to *α*_1_ acid glycoprotein has been reported to reduce the availability of the drug *in vivo*, with a demonstration of resistance *in vitro* (Gambacorti-Passerini *et al*, 2002). Low levels of imatinib have been reported in cerebrospinal fluid (Petzer *et al*, 2002), suggesting that this may be a ‘sanctuary’ site for residual disease.

Increased levels of BCR-ABL kinase, related to genomic amplification of the *BCR-ABL* gene, or increased levels of expression has been observed. Importantly, an *in vitro* model demonstrated that exposure to low levels of imatinib promoted the development of genomic amplification, whereas effective drug levels did not (Le Coutre *et al*, 2000).

Finally, mutations in BCR-ABL may impair imatinib binding. This mechanism has perhaps been most widely studied and appears to be the most common mechanism of resistance in clinical practice (Gorre *et al*, 2001; Branford *et al*, 2002; Hochhaus *et al*, 2002; Shah *et al*, 2002; Branford *et al*, 2003).

To study the frequency of point mutations in *BCR-ABL*, CML patients were screened with sequencing of the *ABL* kinase domain (Branford *et al*, 2003). All had received at least 6 months of imatinib therapy and were studied regardless of response. Mutations were found in 27 out of 144 patients and were associated with acquired resistance in 89% and with shortened survival.

In contrast, point mutations in *BCR-ABL* are not commonly seen in primary refractory patients. In a separate study (Branford *et al*, 2002), only one out of 10 CML patients with primary resistance to imatinib therapy had a *BCR-ABL* mutation, suggesting that a different mechanism is responsible in this setting.

### Are all mutations equally important?

There are limited data regarding the clinical significance of each individual mutation, but overall, their emergence in imatinib resistance indicates an association with poor prognosis. In CML patients treated with imatinib, emergence of mutations at amino-acid positions 250–255, which form the adenosine triphosphate-binding loop (P-loop), carried an especially poor prognosis with 12 of 13 (92%) dying at a median of 18 weeks from the detection of the mutation (Branford *et al*, 2003). Among 14 patients with non-P-loop mutations only three (21%) died, with a similar follow-up from the time of detection.

The *in vitro* significance of individual mutations can be assessed more readily. Methods include inhibition of substrate phosphorylation and inhibition of cell culture. Reported IC_50_ values of clinically detected mutations are presented in Table 2

**Table 2:** IC_50_ values for BCR-ABL mutations

	**IC50 (*μ*M imatinib)**				
**BCR-ABL type**	**Corbin (2003)**	**Azam (2003)**	**Hochhaus (2002)**	**Shah (2002)**	**Von Bubnoff (2002)**
Wild-type ABL			0.025		
Wild-type BCR-ABL	0.5	0.6		0.6	>0.1<0.5
M244V	1.6	3.1			
M244I		1.4			
G250E	4.5	>20		>10	
Q252H	2.6	2.9			
Y253H	>17.7	17.7	3.7		>10
Y253F	5.0		1.8		
E255V	>17.7		>5		>10
E255K	7.5	12.1	>5	>10	>10
F311L	0.7	1.3			
T315I	>17.7	>20		>10	>10
T315S		3.8			
F317L	1.3	2.3		7.5	
M351T	1.5	4.9		4.4	
M351I		1.6			
E355G	2.0			2.4	
F359V	1.4				
V379I	1.0				
L387M	1.1				
H396P	4.3				>1.0<5.0
H396R	5.4				

. These values are derived from *in vitro* cell lines engineered to express a specific mutant *BCR-ABL* gene. For comparison, the mean plasma trough concentration of imatinib 400 mg daily is 1.46 *μ*M (Corbin *et al*, 2003). CML mutant clones with an IC_50_ close to or below this value should be more likely to respond well to dose escalation.

Gambacorti-Passerini *et al* (2003) divide the BCR-ABL mutations into groups according to the functional regions of the molecule where they occur. In the first group, mutations such as T315I are in the imatinib-binding site. Other mutations involve the P-loop (e.g. E255 K) and presumed regulatory regions of the enzyme (e.g. M351T) remote from the imatinib-binding site.

Heinrich *et al* (2002c) suggest a similar classification of the KIT-activating mechanisms of GIST. The major group of abnormalities involves regulatory portions of KIT, so the imatinib-binding region is unaffected. Hopefully, a better understanding of the *C-KIT* mutations present at diagnosis will help optimise both patient selection and tailoring of therapy. As may occur in the accelerated phase of CML, they also present evidence for the acquisition of additional karyotypic abnormalities in more advanced GIST. Such abnormalities may be predicted to augment the KIT-dependent transformation or confer KIT-independent malignant potential. However, the precise molecular mechanisms underlying acquired imatinib resistance in GIST are yet to be elucidated.

Unlike GIST, in systemic mastocytosis activation of KIT is typically associated with point mutations in the enzymatic region of the molecule. The most common of these mutations (D816 V) shows no response to imatinib *in vitro* (Zermati *et al*, 2003), while the proliferation of normal mast cells containing wild-type *C-KIT* was significantly inhibited. It is thought that the D816V mutation interferes with the interaction of imatinib and its KIT binding site. This finding is analogous to T315I in CML, which disrupts the noncovalent interactions essential for the binding of imatinib to the BCR-ABL kinase.

In idiopathic hypereosinophilic syndrome (Cools *et al*, 2003), there is another parallel to the CML experience in that acquired drug resistance was observed in one patient with emergence of a point mutation in *PDGFRA-FIP1L1*, again showing structural analogy to the T315I mutation of *BCR-ABL*. When the IC_50_ of this mutation was tested, it was found to show a 3-log increase from wild type.

### When do resistant mutations arise?

It seems that at least in some cases of CML, mutations conferring resistance are in fact present in a small number of leukaemic cells prior to treatment and are positively selected by imatinib therapy. Interpretation of studies investigating this problem is complicated by the use of different methodologies with varying sensitivity. However, four patients with primary cytogenetic refractoriness had *BCR-ABL* mutations identified using direct cDNA sequencing (Roche-Lestienne *et al*, 2002). Retrospective analysis of pretreatment specimens using allele-specific oligonucleotide PCR (ASO-PCR) was able to demonstrate low levels o000000f mutant cDNA in all four cases, indicating clonal selection. Similarly, von Bubnoff *et al* (2002) noted an increase in the proportion of mutant mRNA with sequential monitoring of relapsing patients.

Further evidence in support of this hypothesis comes from the finding that the duration of disease prior to commencement of imatinib is a significant predictor of resistance-conferring mutations (Branford *et al*, 2003). In a group of 144 patients classified according to disease phase at commencement of imatinib, no early chronic phase patients had detectable mutations, while 22% of late chronic phase patients carried mutations. Furthermore, patients who started imatinib therapy more than 4 years from diagnosis had a higher incidence of mutations (41%) than those treated within 4 years (9%).

### Can imatinib cure CML?

Despite excellent initial responses to imatinib, it is unclear whether long-term control of chronic phase CML will be achieved with monotherapy. Although imatinib has been shown to promote apoptosis, there are *in vitro* data indicating that primitive Ph+ stem cells not undergoing mitosis remain viable following exposure to the drug (Graham *et al*, 2002). Such cells could provide a therapy-resistant pool responsible for late relapse.

In advanced phases of CML, there may be secondary oncogenic abnormalities (e.g. *P*53 mutations) that change the biologic behaviour of the CML clone. Emergence of BCR-ABL-independent pathways supporting proliferation or resistance to apoptosis will inevitably diminish the efficacy of imatinib monotherapy.

### Can imatinib combination therapy overcome resistance?

There is significant *in vitro* evidence indicating synergism of combinations of imatinib with conventional cytotoxics and newer biological therapies. Inhibition of heat shock protein, hsp90, and histone deacetylase results in degradation of BCR-ABL and induction of apoptosis in imatinib-resistant CML cell lines and shows synergism with imatinib (Schad *et al*, 2002; Nimmanapalli *et al*, 2003). Targeting alternative pathways of signal transduction, for example with farnesyl transferase inhibitors, also shows promise in imatinib-resistant CML cell lines (Hoover *et al*, 2002; Topaly *et al*, 2002).

Following on from *in vitro* studies, there are a number of preliminary reports of Phase I/II trials in imatinib-resistant CML using imatinib with gemtuzumab anti-CD33 monoclonal antibody (Sallah *et al*, 2002), imatinib with farnesyl transferase inhibitor, lonafarnib (Cortes *et al*, 2002a), and imatinib with cytotoxics (Mauro *et al*, 2002; Fruehauf *et al*, 2003; Maloisel *et al*, 2003). Overall, the main toxicity of combination therapy was increased myelosuppression, although this was generally well-tolerated.

In newly diagnosed chronic phase CML, trials combining imatinib and interferon *α* (Baccarani *et al*, 2002; Hochhaus *et al*, 2003) or imatinib and cytarabine (Cornelissen *et al*, 2002; Gardembas *et al*, 2002) are in progress. It seems likely that combination approaches will lead to a lower incidence of resistance, but this remains to be tested formally.

## IMPLICATIONS FOR CANCER THERAPY

The use of imatinib in CML has been a striking success and has spurred an increase in research to develop molecular-based therapies in a range of conditions. What has this experience taught us about the use of kinase inhibitors in cancer therapy? Firstly, it is worth noting that some observations may be unique to CML. Most cancers are driven by multiple aberrant pathways and blockade of a single pathway may not represent effective monotherapy. Thus, the remarkable activity of imatinib as monotherapy may not be observed in most cancer settings. However, some key observations are likely to have broader implications:

1. Experience in CML, and more recently in GIST and myeloproliferative disorders, indicates the importance of identifying a molecular target that can be inhibited and that also provides a critical transforming signal for tumour cells. This needs to be distinguished from tumours that simply express a potential target kinase, but are not critically dependent upon the kinase pathway.
2. Blockade of a kinase that provides a significant growth signal in malignant cells may nullify the proliferative advantage of a tumour, but may not induce apoptosis. In CML, the amount of leukaemia present in the blood and marrow falls rapidly over the first few months of imatinib therapy, but residual leukaemia is usually still detectable with longer follow-up. This is the predicted pattern of response for an agent that blocks proliferation but does not induce cell death in leukaemic stem cells. The stem cell population may remain relatively intact because they are generally quiescent and not dependent on overactive kinase activity for survival. Combination therapy with a second agent capable of inducing apoptosis, or inducing cell division in quiescent cells may prove highly effective.
3. Molecular diagnostics are playing an increasingly important role in the assessment of malignancy. At least in myeloproliferative disorders, and perhaps also in GIST, there is potential for patients with a similar clinicopathological presentation to harbour differing genetic abnormalities involving related tyrosine kinase pathways. This emphasises the need for accurate genetic diagnosis wherever possible. Molecular diagnosis will also improve our capacity to monitor disease response.
4. Ideally, patients should be selected on the basis of *in vitro* testing to establish that their tumour is effectively targeted. Assessment of a kinase-dependent malignancy could include a screen for kinase mutations or polymorphisms that prevent binding of the kinase inhibitor and/or determination of the IC_50_. This would enable both appropriate use of the kinase inhibitor and identification of patients who will benefit from dose escalation or combination therapies. Optimal dosing should be based on extensive pharmacokinetic studies with verification of optimal target blockade. Where tumour tissue is available, PCR-based detection of drug-resistant mutations should be a component of monitoring, so that necessary changes to therapy may be initiated early.
5. The development of acquired resistance is a significant concern in the CML setting, with over 20 possible mutations in BCR-ABL capable of leading to resistance. This illustrates the potential limitation of monotherapy using a highly specific kinase inhibitor that is likely to be relevant for many cancers. A similar observation of resistance due to kinase point mutation has been reported in a patient receiving imatinib for idiopathic hypereosinophilic syndrome.
6. In CML, imatinib has its highest response rate when used soon after diagnosis, while resistant kinase mutations increase in frequency with disease duration and advanced phase of disease. Early and effective kinase inhibition may also be crucial to optimise treatment outcomes in other kinase-dependent malignancies.
7. Most importantly, careful elucidation of the cause of resistance in CML has led to detailed understanding of how the kinase works and how it interacts with inhibitors. This understanding has contributed to the development of new BCR-ABL inhibitors designed to be less susceptible to inactivation by a single amino acid substitution as well as providing an additional rationale for combination therapy. Similar detailed analysis of the cause of resistance with other kinase inhibitors is likely to be equally valuable.
